# Identification and Classification of Rare Variants in *NPC1* and *NPC2* in Quebec

**DOI:** 10.1038/s41598-021-89630-5

**Published:** 2021-05-14

**Authors:** Lahoud Touma, Marjorie Labrecque, Martine Tetreault, Antoine Duquette

**Affiliations:** 1 Départment de Neurosciences, Faculté de Médecine, Université de Montréal, CRCHUM – 900, rue Saint-Denis, Pavillon R, Montréal, QC H2X 0A9 Canada; 2 Service de Neurologie, Département de Médecine, Unité de Troubles du Mouvement André-Barbeau, Centre Hospitalier de L’Université de Montréal (CHUM), Montreal, Canada; 3 Service de Médecine Génique, Département de Médecine, Centre Hospitalier de L’Université de Montréal (CHUM), Montreal, Canada; 4 Centre de Recherche du Centre Hospitalier de L’Université de Montréal (CRCHUM), Montreal, Canada

**Keywords:** Neuroscience, Diseases of the nervous system, Genetics of the nervous system

## Abstract

Niemann–Pick disease type C (NPC) is a treatable autosomal recessive neurodegenerative condition which leads to a variety of progressive manifestations. Despite most cases being diagnosed at a young age, disease prevalence may be underestimated, especially in adults, and interpretation of *NPC1* and *NPC2* variants can be difficult. This study aims to identify potential pathogenic variants in a large cohort of healthy individuals and classify their risk of pathogenicity to assist with future interpretation of variants. The CARTaGENE (CaG) cohort was used to identify possible variants of *NPC1* and *NPC2*. Nine-hundred and eleven RNA samples and 198 exome sequencing were screened for genetic variants through a bio-informatic pipeline performing alignment and variant calling. The identified variants were analyzed using annotations for allelic frequency, pathogenicity and conservation scores. The ACMG guidelines were used to classify the variants. These were then compared to existing databases and previous studies of NPC prevalence, including the Tübingen NPC database. Thirty-two distinct variants were identified after running the samples in the RNA-sequencing pipeline, two of which were classified as pathogenic and 21 of which were not published previously. Furthermore, 46 variants were both identified in our population and with the Tübingen database, the majority of which were of uncertain significance. Ten additional variants were found in our exome-sequencing sample. This study of a sample from a population living in Quebec demonstrates a variety of rare variants, some of which were already described in the literature as well as some novel variants. Classifying these variants is arduous given the scarcity of available literature, even so in a population of healthy individuals. Yet using this data, we were able to identify two pathogenic variants within our population and several new variants not previously identified.

## Introduction

Niemann–Pick disease type C (NPC) is a rare, autosomal recessive neurodegenerative condition which leads to a variety of progressive neurological and non-neurological manifestations. NPC is estimated to affect 1 in 100,000–120,000 live births^1^. It is caused by mutations of the *NPC1* gene in 95% of cases while mutations of *NPC2* account for the other 5%. The spectrum of clinical presentation is wide with different onsets which have been classified as: perinatal (shortly before and after birth, 3–12% of cases), early infantile (3 months to < 2 years, 3–37% of cases), late infantile (2 to < 6 years, 21–39% of cases), juvenile (6 to < 15 years, 21–54% of cases), and adult (15 years and greater, 5–27% of cases)^2,3^. In the majority of cases, patients first develop liver, spleen or lung involvement with a high variability of disease severity. These are then followed by progressive neurological symptoms classically presenting with cerebellar ataxia, vertical supranuclear gaze palsy, gelastic cataplexy, seizures and eventually dementia^4^. While studies of *NPC2* are easily interpretable, *NPC1* is highly polymorphic where variants of unknown significance (VUS) are common, with one third of published variants being classified as VUS on ClinVar^3,5^.

NPC is one of the few degenerative ataxias for which a treatment is currently available. Miglustat (*Zavesca*) has been approved in several countries for treating progressive neurological complications of NPC^6^. The drug has been shown to slow progression of neurological manifestations in children without severe neurological symptoms when initiating therapy, as shown by nonsignificant improvement in horizontal saccadic eye movement velocity in a preliminary open-label randomized controlled trial^7^. Miglustat was also associated with improved swallowing function and decreased aspiration risk in observational studies^8,9^.

Despite most cases being diagnosed at a young age, the adult-onset form can be more insidious and often manifests with neuropsychiatric disturbances. The diagnosis is based on clinical evaluation and history with biomarker screening. Several blood-based biomarkers can be used to assist with diagnosis including oxysterols, lysosphingolipids and bile acid metabolites. Consensus guidelines still recommend completing the workup of suspected cases with two additional diagnostic methods: filipin staining of unesterified fibroblasts or molecular testing^6,10^. The former requires a skin biopsy with a specialized laboratory, but can be inconclusive in 15% of cases without molecular testing^11^. Molecular testing is more practical but can also be inconclusive in up to 15% of cases mainly due to VUS and the absence of allele segregation studies^2^. The assessment of these variants will require additional data input from various laboratories to allow for more specific classification.

CARTaGENE (CaG) is a cohort of healthy individuals living in Quebec^12^. The cohort contains a total of 43,000 individuals, including 55% of women, ranging between 40 and 69 years of age. The recruitment for this cohort started in 2010 and participants have been followed up to this date. Genetic data is available for some of these individuals in the form of RNA as well as exome sequencing. This data can thus be used to screen for potential variants in a healthy pool of the population.

The study aim was to identify potential pathogenic variants in *NPC1* and *NPC2* in healthy individuals from the CaG cohort and classify their risk of pathogenicity using the *American College of Medical Genetics and Genomics* (ACMG) guidelines revised version of 2015^13^ to help assist with the future interpretation of variants by providing useful additional information derived from large databases.

## Materials and methods

### Initial data

This study was based on a random sample from the CaG cohort, which was representative of the regional distribution of the Quebec population. Data acquisition was made from 911 individuals for the RNA-sequencing (RNA-seq) and 198 individuals for exome-sequencing (exome-seq). 93 of these individuals were in both RNA-seq and exome-seq. A bio-informatic pipeline was therefore used to analyse a total of 1016 individuals. Baseline characteristics as well as screening medical questionnaires were obtained for each participant from the CaG database. Informed consent was previously obtained by CaG researchers for all study participants. The Sample and Data Access Committee (SDAC) of CaG approved the use of the genetic and baseline characteristics for our study. All genetic and bioinformatic analysis were carried out in accordance with relevant guidelines and regulations. Our protocol was approved by our institution’s research ethics board (CR-CHUM REB, Project 18.116).

### Bio-informatic pipeline

The FASTQ files were aligned to the reference genome (Hg19) using BWA for exome sequencing and STAR for RNA-sequencing^14,15^. In both cases, variant calling was performed with GATK and annotated using ANNOVAR and custom scripts^16,17^. The bioinformatic pipeline in place allows the detection of single nucleotide variants (SNV) either non-synonymous, splice junction or synonymous, multi-nucleotide variants (MNV) and indels.

Since NPC1 (NM_000271) and NPC2 (NM_006432) genes are located on chromosome 18 or 14 respectively, we only extracted variants on those chromosomes for each sample. We also added information from the NP-C database (NPC-db2) made by the University of Tübingen, using a custom Python script. The database was last searched in July 2019. When a variant was found in the NPC-db2, its pathogenicity classification based on their criteria was added to the resulting file.

### Analysis

As we were identifying rare variants, common variants (defined as > 1%) found in dbSNP, 1000 Genomes, Exome Variant Server, GnomAD and internal databases were filtered out. Non-synonymous, putative splicing variants and coding indels were prioritized. More specifically, we set a threshold for a CADD score higher than 15, a Polyphen2 score higher than 0.75 with a score of one being very likely pathogenic, a SIFT score, that was reversed to match the Polyphen2 score, with the same criteria as Polyphen2 to filter the variants^18–22^. For the conservation scores, we identified the variants with a score higher than 500 for Phast cons and higher than 5 for GERP. Phast cons ranges between 0 and 1000 and the GERP score between − 12.3 and 6.17.

### Classification

The ACMG 2015 revised guidelines were used to classify the different variants. The classification uses five distinct categories: benign, likely benign, uncertain significance, likely pathogenic and pathogenic. Each variant was analyzed using the ACMG criteria except the ones requiring segregation and laboratory data which were not available for our dataset. We extracted the NPC-db2 classification for each variant. Thereafter, the variants were searched on ClinVar for previous classification by other groups, using the ACMG criteria. Classifications based on other sets of criteria were not included in our tables. Finally, we searched the largest published study on NPC variants by Wassif et al*.* for identical variants already identified in their results. Previously unreported variants will be submitted to the ClinVar public database.

## Results

### Baseline characteristics

The total sample size for both RNA-seq and exome-seq was 1016 patients and 2032 chromosomes. Clinical data was available for 1004 patients (Table 1). Females represented 51% of our population. The highest represented ethnicity was white from European descent (91.5%). The majority of these individuals were employed (64.3%). Age ranged from 44 to 69, with 42% of patients in the 40–49 range. This sample has a similar distribution as the general CaG cohort (Table 1). Additionally, the sample is also representative of the Quebec population based on the most recent epidemiological data^23^.

**Table 1:** Baseline characteristics.

	Sample (%)	CaG (%)	Quebec (%)
**Female**	50.6	55	50.0
**Age**			
40–49	42.3	42	31.3
50–59	29.0	37	32.6
60–69	28.7	25	36.1
**Ethnicity**			
White (European Descent)	91.5		87.0^b^
Arab	2.2		2.7^b^
Black (African or Caribean descent)	1.7		4.0^b^
**Employed**	64.3	67	62.4
**Highest level of education**			
University	37.1	45	31.2^a^
College	49.8	32	45.2^a^

^a^Data for age range of 35–64 in the Quebec population (Banque de données des statistiques officielles sur le Québec 2015).

^b^Data for overall province of Quebec (Statistics Canada 2016).

### RNA-seq

**Figure 1:**
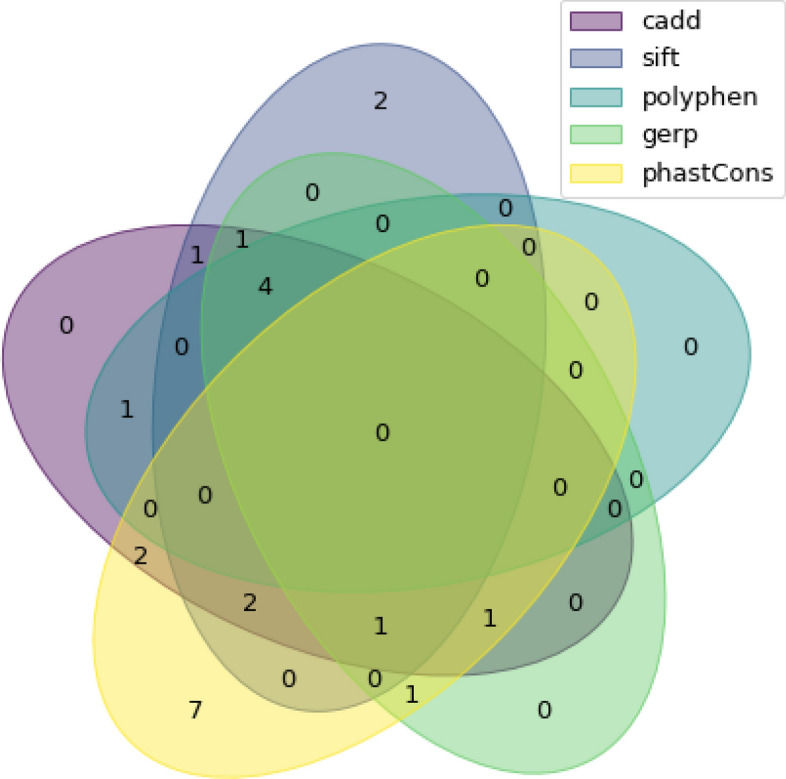
Venn diagram of included variants from RNA-seq for each bioinformatic filter.

Our study identified 32 unique rare variants from the 911 RNA samples that were run in the bio-informatic pipeline (Table 2, Fig. 1). Each variant was only present in one chromosome, for an allele frequency of 0.05% in our population. None of the study participants were heterozygous for two rare variants. Twenty of these variants were non-synonymous SNVs while the others were frameshift deletions. Among these variants, two were classified as pathogenic. Indeed, the p.Ile1061Thr is a known protein change that leads to a change from isoleucine to threonine. This variant has been described as causative of NPC in 15–20% of disease alleles in the United States and Europe. Additionally, biological studies have shown that this missense change affects proper protein localization and causes proteasomal degradation in cell culture. Another pathological variant, p.Pro543Leu, has been identified in 1 homozygous and 4 compound heterozygous individuals with symptomatic disease^24^. It has previously been reported that this mutation leads to early-infantile form of NPC^25^. Both participants were heterozygous for these mutations and were asymptomatic according to the baseline medical screening obtained from CaG. The remaining twelve variants were indels and all were classified as VUS. Given the limited coverage, these could represent artifacts and their exact significance is difficult to interpret.

**Table 2:** Rare variants in CARTaGENE sample, RNA-seq.

Variants	Variations	Ref	Alt	Protein Change	Gene	ACMG Classification	Classification in NPC-db2	ClinVar (ACMG)	*Wassif *et al
chr18:21121118	Nonsynonymous SNV	C	A	p.V810F	NPC1	3	2	3	–
chr18:21140411	Nonsynonymous SNV	T	C	p.N222S	NPC1	2	1	3	Benign
chr18:21136233	Nonsynonymous SNV	G	A	p.P434S	NPC1	2	1	–	Benign
chr18:21140367	Nonsynonymous SNV	G	A	p.P237S	NPC1	2	1	1	Benign
chr18:21113406	Nonsynonymous SNV	T	C	p.I1223V	NPC1	2	2	–	Benign
chr18:21119839	Nonsynonymous SNV	C	T	p.G911S	NPC1	2	1	–	Benign
chr18:21131617	Nonsynonymous SNV	G	A	p.P543L	NPC1	5	4	5	Probably damaging
chr18:21121386	Nonsynonymous SNV	C	T	p.V753M	NPC1	3	2	3	Benign
chr18:21114442	Nonsynonymous SNV	C	T	p.A1187T	NPC1	3	–	3	–
chr18:21140243	Nonsynonymous SNV	G	A	p.A278V	NPC1	3	–	–	–
chr18:21136410	Nonsynonymous SNV	T	C	p.T375A	NPC1	3	–	–	–
chr18:21134806	Nonsynonymous SNV	T	C	p.N490S	NPC1	3	–	–	–
chr18:21134743	Nonsynonymous SNV	G	A	p.T511M	NPC1	2	2	–	Probably damaging
chr18:21118536	Nonsynonymous SNV	G	A	p.S1004L	NPC1	3	2	–	Probably damaging
chr14:74959920	Nonsynonymous SNV	C	T	p.E20K	NPC2	3	–	–	–
chr18:21136422	Nonsynonymous SNV	C	A	p.V371F	NPC1	3	–	–	–
chr18:21140315	Nonsynonymous SNV	G	T	p.P254Q	NPC1	3	–	–	–
chr18:21136439	Nonsynonymous SNV	G	A	p.S365L	NPC1	3	–	–	Possibly damaging
chr18:21121045	Nonsynonymous SNV	A	G	p.M834T	NPC1	3	2	–	Benign
chr18:21116700	Nonsynonymous SNV	A	G	p.I1061T	NPC1	5	5	5	Benign
chr18:21153473	Frameshift deletion	AT	A	p.N41fs	NPC1	3	–	–	–
chr18:21125100	Frameshift deletion	CA	C	p.F590fs	NPC1	3	–	–	–
chr18:21141470	Frameshift deletion	TC	T	p.D162fs	NPC1	3	–	–	–
chr18:21121129	Frameshift deletion	TC	T	p.D806fs	NPC1	3	–	–	–
chr18:21140314	Frameshift deletion	TG	T	p.P254fs	NPC1	3	–	–	–
chr18:21119811	Frameshift deletion	AC	A	p.V920fs	NPC1	3	–	–	–
chr18:21120443	Frameshift deletion	AT	A	p.I858fs	NPC1	3	–	–	–
chr18:21153486	Frameshift deletion	TC	T	p.D37fs	NPC1	3	–	–	–
chr18:21124431	Frameshift deletion	AG	A	p.A669fs	NPC1	3	–	–	–
chr18:21116757	Frameshift deletion	TG	T	p.H1042fs	NPC1	3	–	–	–
chr18:21114436	Frameshift deletion	CTT	C	p.E1188fs	NPC1	3	–	–	–
chr18:21140211	Frameshift insertion	C	CA	p.A289fs	NPC1	3	–	–	–

^a^Classification 1: Benign 2: Likely benign 3: Uncertain significance 4: Likely pathogenic 5: Pathogenic.

### Comparison with other databases

The NPC-db2 database was searched for identified variants which were also present in our population. Twelve out of the 32 variants identified were also present in their sample. Our classification based on the ACMG criteria was overall very similar to their classification for shared variants. Notably, the p.Pro543Leu protein change was marked as potentially pathogenic in NPC-db2, while we were able to classify it as pathogenic based on previous publications and computational/predictive data. Additionally, we identified 13 variants that were filtered out by our bioinformatic pipeline, but that were present both in our population and in the NPC-db2 database (Table 3). These were all previously classified as benign. The main reason for their exclusion in the pipeline was a high allelic frequency (> 1%).

**Table 3:** Variants in the CARTaGENE RNA-seq sample excluded from the pipeline but identified in NPC-db2.

Variants	Variations	Ref	Alt	Protein Change	Gene	ACMG Classification	Classification in NPC-db2	ClinVar (ACMG)	*Wassif *et al
chr18:21124945	nonsynonymous SNV	C	G	p.M642I	NPC1	1	1	1	–
chr18:21112206	nonsynonymous SNV	C	T	p.R1266Q	NPC1	1	1	1	Benign
chr18:21120444	nonsynonymous SNV	T	C	p.I858V	NPC1	1	1	1	Benign
chr18:21140432	nonsynonymous SNV	T	C	p.H215R	NPC1	1	1	1	Benign
chr18:21140367	nonsynonymous SNV	G	A	p.P237S	NPC1	1	1	1	Benign
chr18:21136233	nonsynonymous SNV	G	A	p.P434S	NPC1	1	1	–	Benign
chr18:21140411	nonsynonymous SNV	T	C	p.N222S	NPC1	1	1	2, 3	Benign
chr18:21148863	synonymous SNV	A	G	p.Y129Y	NPC1	1	1	–	Benign
chr18:21115579	synonymous SNV	G	A	p.L1111L	NPC1	2	1	–	–
chr18:21124335	synonymous SNV	G	A	p.N701N	NPC1	1	1	–	–
chr18:21134772	synonymous SNV	G	A	p.D501D	NPC1	2	1	–	–
chr18:21114440	synonymous SNV	C	A	p.A1187A	NPC1	2	1	–	–
chr18:21124365	synonymous SNV	C	T	p.P691P	NPC1	2	1	–	–

^a^Classification 1: Benign 2: Likely benign 3: Uncertain significance 4: Likely pathogenic 5: Pathogenic.

The variants in the study by Wassif et al*.* were also compared with variants identified in our study (Table 2)^26^. Despite not specifically using the ACMG criteria, we were able to compare their five-level scale of classification to our data. Twelve out of the 32 variants were also classified in their study. One notable difference in classification in the variant p.Ile1061Thr was probably due to a mistake in their table as they present it as benign, while describing it as on of the most common pathogenic variant in their text^26^. Moreover, five variants that were in both our databases were excluded by our pipeline. Once again, these were classified as benign and the main reason was a high allelic frequency (> 1%).

### Exome-seq

**Figure 2:**
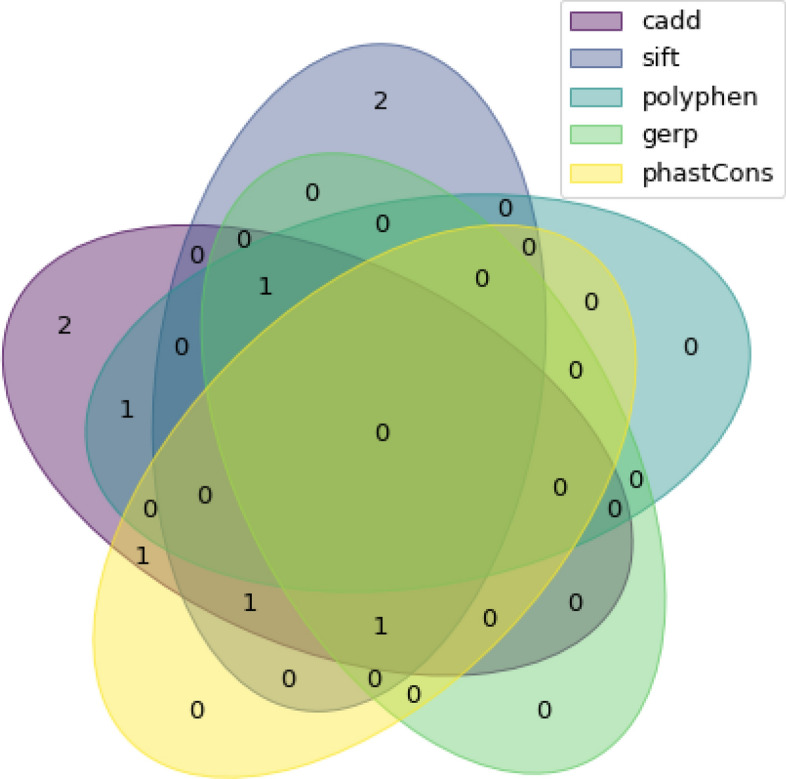
Venn diagram of included variants from exome-seq for each bioinformatic filter.

Exome sequencing was performed for 198 individuals, composed of 93 individuals for whom we also had the RNA-seq and 105 new individuals for whom we only had exome-seq. Overall, 19 unique variants were identified in the samples, 4 of which were also present in the RNA-seq (Fig. 2). In participants for whom both RNA-seq and exome-seq data were available, the exact same sequence variants were found using both methods. After filtering by the bioinformatic pipeline, 10 variants were identified. Four of those were already found in our RNA-seq, including one classified as pathogenic (Table 4). The six other variants were found in individuals for which we only had exome data. These included two variants in splicing regions, one of which was causative of disease in previous publications^24,27^. The participant was heterozygous for this variant and was asymptomatic according to baseline medical screening obtained from CaG. The RNA-seq for these individuals were not available to confirm the presence of abnormal splicing.

**Table 4:** Rare variants in CARTaGENE sample, exome-seq.

Variants	Variations	Ref	Alt	Protein Change	Gene	ACMG Classification	Classification in NPC-db2	ClinVar (ACMG)	*Wassif *et al
chr18:21118536	nonsynonymous SNV	G	A	p.S1004L	NPC1	3	2	4	Probably damaging
chr18:21134806	nonsynonymous SNV	T	C	p.N490S	NPC1	3	–	–	–
chr18:21121118	nonsynonymous SNV	C	A	p.V810F	NPC1	3	2	3	–
chr18:21118618	nonsynonymous SNV	C	T	p.V977I	NPC1	3	–	–	–
chr18:21116700	nonsynonymous SNV	A	G	p.I1061T	NPC1	5	5	5	Pathogenic
chr18:21115615	nonsynonymous SNV	T	C	p.I1099V	NPC1	3	–	–	–
chr14:74947404	splicing	C	T		NPC2	3	–	3	Strong negative
chr14:74953027	splicing-extended	C	T		NPC2	3	–	3, 5	–
chr14:74959920	nonsynonymous SNV	C	T	p.E20K	NPC2	3	–	–	–
chr14:74951269	nonsynonymous SNV	T	C	p.K71R	NPC2	3	–	–	Probably damaging

^a^Classification 1: Benign 2: Likely benign 3: Uncertain significance 4: Likely pathogenic 5: Pathogenic.

## Discussion

Our study evaluated rare variants in *NPC1* and *NPC2* genes in a sample from the Quebec population in Canada. This population is unique given the important founder effect from French colonisation in the early seventeenth century^28^. We had 911 RNA samples and 198 exome samples, with an overlap of 93 individuals. This study presents new variants that have not been previously described in the literature. In addition, known variants were reclassified based on the most recent literature. In fact, we classified each individual variant based on the ACMG 2015 guidelines and compared them to the NPC-db2 database and previously published studies on such variants. The majority of identified variants were of uncertain significance, likely benign or benign. However, we have also identified some likely pathogenic and pathogenic variants in heterozygous individuals.

To select rare variants in our population, we used a pre-specified bioinformatic pipeline. The variants were filtered based on their allelic frequency, with a coverage of at least 15% and where the alternative base was supported at least twice. This ensured that the classification would be applied to the most pertinent variants in our sample. We then focused our analysis on indels and non-synonymous variants, which were more likely to lead to pathogenic mutations. Additional variants were manually identified by comparing all variants present in our sample to those in the NPC-db2 database. These were filtered out from our pipeline according to the aforementioned criteria but were still classified by our laboratory because they were coincidentally present in another study population. The main reason for exclusion was allelic frequency > 1%.

With the increased use of genetic testing and the identification of more variants, it has become essential to apply rigorous classification in clinical genetic testing^29^. The set of criteria must be evidence-based, standardized and objective^30^. The ACMG 2015 guidelines, used in our study, have been largely used and therefore allow for easier comparison with previous publications. Individual laboratories also share their own classification in large databases (including ClinVar), but it is difficult to compare with their conclusions as the set of criteria is different. Thus, we have only compared our classification with published literature using the same set of criteria.

The CARTaGENE database encompasses a large sample of genomic data, but also baseline information based on detailed questionnaires. Answers included demographics, socioeconomic status, education and medical surveys. Given the possible adult-onset of NPC, we searched for potential symptoms in the questionnaires of patients with pathogenic or likely pathogenic variants. None of the identified individuals presented symptoms suggestive of the disease, as we had expected given the heterozygosity of the alleles. These pathogenic variants will allow us to estimate the carrier frequency in the Quebec population.

Our study has several potential limitations. First, our dataset is based on a relatively small sample size of 1016 individuals. However, given the important founder effect in our Quebec population pool, genetic variation is relatively lower when compared to other populations^31^. Second, we did not perform any functional biology experiments which limits our ability to classify some of these mutations based on functional criteria. Third, no segregation data was available in the database, which can often provide strong evidence for a benign variant in a new mutation.

In brief, this study analyzed variants in the *NPC1* and *NPC2* genes from a representative sample of the Quebec population. The results described novel variants that were not previously described in the literature. In addition, known variants were reclassified using the ACMG guidelines. Despite identifying pathogenic or likely pathogenic variants, the individuals were heterozygous and asymptomatic based on baseline questionnaires. Classifying these variants is arduous given the scarcity of available literature, even so in a population of healthy individuals, leading to a large proportion of variants of uncertain significance. Using this data, we were able to identify three pathogenic variants within our population and several new rare variants in *NPC1* and *NPC2* which had not previously been identified. This additional information should help clinicians interpret the pathogenicity of variants identified in these two genes moving forward.

## Data Availability

Data was provided by the CARTaGENE database from a sample from the Quebec population. The data generated or analyzed during this study are included in this published article and its supplementary information files.

## Code availability

We used publicly and freely accessible codes, referenced throughout our method section. Our custom Python scripts are available if necessary.
